# Young children conform more to norms than to preferences

**DOI:** 10.1371/journal.pone.0251228

**Published:** 2021-05-26

**Authors:** Leon Li, Bari Britvan, Michael Tomasello

**Affiliations:** 1 Department of Psychology and Neuroscience, Duke University, Durham, North Carolina, United States of America; 2 Department of Developmental and Comparative Psychology, Max Planck Institute for Evolutionary Anthropology, Leipzig, Germany; Middlesex University, UNITED KINGDOM

## Abstract

As members of cultural groups, humans continually adhere to social norms and conventions. Researchers have hypothesized that even young children are motivated to act conventionally, but support for this hypothesis has been indirect and open to other interpretations. To further test this hypothesis, we invited 3.5-year-old children (*N* = 104) to help set up items for a tea party. Children first indicated which items they preferred but then heard an informant (either an adult or another child) endorse other items in terms of either conventional norms or personal preferences. Children conformed (i.e., overrode their own preference to follow the endorsement) more when the endorsements were framed as norms than when they were framed as preferences, and this was the case whether the informant was an adult or another child. The priority of norms even when stated by another child opposes the interpretation that children only conformed in deference to adult authority. These findings suggest that children are motivated to act conventionally, possibly as an adaptation for living in cultural groups.

## Introduction

To become functioning members of a culture, young children must learn about not only physical reality but also social reality. This poses a considerable learning challenge, as many aspects of social reality, such as norms, conventions, and rituals, are causally opaque with no obvious instrumental functions [1–4]. Still, despite their causal opacity, young children need to learn and perform them for reasons such as affiliating and identifying with members of their culture [4, 5]. Along these lines, several researchers have advanced an intriguing hypothesis: that young children are motivated to act conventionally [4, 6–8]. The hypothesis, to put it more concretely, is that when young children perceive that a certain action is conventional within their cultural group, they will be motivated to perform that action simply out of a desire to act conventionally—independent of other possible motives for performing that action.

This hypothesis is significant because it helps explain how human groups are able to preserve and transmit cultural practices over generations. Cultural practices would not persist, after all, if younger generations were not motivated to acquire them. To date, the hypothesis that young children are motivated to act conventionally has received support from several empirical studies. In this paper, we aim to add to this literature by taking a closer look at whether young children’s motivation to perform conventional actions is indeed independent of other possible motives for performing such actions. This inquiry is warranted because, as we describe below, previous studies on this issue did not completely rule out other possible explanations for why children may be motivated to perform conventional actions.

On the basis of previous studies, researchers have argued that children interpret certain social cues to be indications that actions are conventional and thus important to adopt [4]. Intentionality has been posited to be one such cue. Even before two years of age, children imitate intentional actions more often than they imitate unintentional actions [9]. Additionally, children who observe an actor performing an action intentionally, as opposed to unintentionally, protest more when a puppet performs the action in a different way [10]. Children also protest when actors omit causally unnecessary steps of actions that the children previously saw demonstrated with the needless steps, even when the children know that the steps are unnecessary [3]. Both of these protest findings [3, 10] suggest that children consider others’ intentional actions to be representative of the socially normative way to act. In addition, the conduct of a majority has been posited to be another cue of conventionality. When observing actors operate a device to obtain a reward, children conform more to a method used by multiple individuals than to a method used by only one individual [11]. By “conform,” it was meant that the children actually switched from their own method of operating the device to adopt the newly observed method, potentially out of a desire to be like the group [11].

However, children may be motivated to adopt actions that they see others performing intentionally or in a majority for reasons other than seeking to act conventionally per se. Regarding intentionality cues, children may potentially seek to imitate another person’s intentional actions out of a desire to affiliate with the person individually. From an early age, children imitate others’ actions as a means of socially bonding with others, such as in the context of preverbal protoconversations [12, 13]. Social bonding is more likely to be achieved by imitating the intentional, as opposed to unintentional, actions of others. As such, even if children adopt others’ intentional actions more often than they adopt others’ unintentional actions, this could be due to a desire to affiliate with others, not necessarily a desire to act conventionally.

As for majority cues, the children in [11] may have inferred that the method used by multiple individuals was more instrumentally effective at obtaining the desired reward compared to the method used by only one individual. After all, perhaps the very reason the former method was more widely used than the latter method was because it was more effective at obtaining rewards. As such, the children may have adopted the method used by the majority not because they wanted to act conventionally but simply because they wanted to increase their chances of obtaining the reward. Overall, previous studies did not rule out possible alternative interpretations for why children may follow intentionality or majority cues. Children may follow these cues not because they seek to act conventionally per se but rather because they seek to affiliate with others or obtain rewards. These issues may be addressed by modeling actions with no instrumental functions and employing linguistic cues, not intentionality or majority cues, to signal conventionality.

One straightforward way to linguistically signal conventionality is to state a rule [14]. But rules may not be an ideal operationalization of conventionality because the source of a rule’s normative force may be unclear to children. It has been argued that norms have two aspects: generality and force [15]. Whereas generality refers to a norm’s widespread applicability to all the members of the group, force refers to group members’ desire that a norm be followed and their willingness to enforce the norm on others. Young children may sometimes experience adults imposing arbitrary rules seemingly based only on their own discretion (e.g., “Does Mom want me to clean my room because cleanliness is a general expectation or only because she herself likes it clean?”). As such, rules may sometimes appear to children to have force stemming not from conventionality but rather from the authority of individual adults.

In recent studies using more subtle linguistic cues, children imitated an actor’s method of making a necklace with higher fidelity when the activity was linguistically framed as conventional than when it was framed as instrumental [1, 2]. However, these studies still used an instrumental context (necklace making) and only examined imitation, not conformity, since the children did not have a prior method that the linguistic framing overrode. In our study, therefore, we first assessed children’s preferences and then examined whether they would conform to another person’s different choice—and whether the conformity would be greater when the choice was linguistically framed as a conventional norm than when it was framed as a personal preference.

Children’s ability to distinguish between norms and preferences has been investigated in previous research. One study examined children’s relative consideration of information about rules versus information about others’ preferences when predicting others’ behaviors and mental states [16]. In this study, children from 4 to 5 years of age weighed information about rules more highly, whereas children from 7 to 8 years of age weighed information about preferences more highly [16]. Another study found that children improved with age at distinguishing between group norms and their own preferences [17]. But this study examined older children (9-year-olds and 13-year-olds) and also focused only on children’s judgments of hypothetical stories, not their behaviors [17]. We aimed to recruit the youngest children who would still be linguistically competent enough to comprehend our linguistic cues. The age of 3.5 seemed suitable, as this was approximately the earliest age at which conventional linguistic framing had been shown to have an effect in previous research [2].

We invited 3.5-year-olds to help set up a pretend tea party, a context without instrumental aims, and varied whether an informant endorsed tea party items, such as cups, in terms of either norms or preferences. In the norm condition, we avoided using prescriptive cues of normative force (e.g., “one should use this cup” or “the rule is to…”), instead relying on descriptive cues of generality (e.g., “we always use…”). This enabled more confidence that children’s conformity to norms represented a respect for conventionality, not just a respect for the force stemming from the authority of the messenger. Preferences were chosen as a control to norms because they may invoke a motivation to conform to affiliate with the informant individually but not necessarily a motivation to act conventionally. Thus, if children conform more to norms than to preferences, this would imply that children seek to act conventionally above and beyond merely seeking to affiliate with the informant individually.

One methodological concern was that if we used an adult model, as most other studies have done, then children might only conform out of a deference to adult authority, not out of a genuine respect for conventionality. To address this concern, we used models of two ages. For some children, the informant who expressed norms and preferences was an adult, whereas for other children, the informant was a 6-year-old child. By using these two models, we could test whether our hypothesized effect (greater conformity to norms than to preferences) would hold even when children did not perceive the model as having authority.

## Method

### Participants

Participants were 3.5-year-olds (*N* = 104, *M* age = 42 months, *SD* = 2, range: 39 to 45; 53 girls) from the Southeastern United States and were recruited via phone calls to parents in our university’s database of local birth records. Because we were employing a newly invented procedure, which had never been used in previous research, we had no basis for estimating an effect size or the sample size needed to detect it. However, a general rule of thumb for factorial designs, such as the 2 x 2 design used in this study, is to obtain at least 24 participants per cell. Our recruitment efforts enabled us to finish data collection with 26 participants per cell.

Participants’ families were mostly white (75% white, 9% black, 2% Asian, 14% biracial or other) and middle-class (over 75% had family incomes exceeding $60,000). Additional children were recruited but excluded from the final sample due to procedural error (9), parental influence (4), lack of English (2), insufficient age (1), the child being excessively distracted and not focused on the activity (1), the child misunderstanding the activity (2), or the child not engaging in the activity and thus not providing any usable data (2). Children were given a toy, book, or T-shirt for participating. This study was approved by the Institutional Review Board of Duke University on March 23, 2018. The procedure was conducted with the written and informed consent of the parents or guardians of the minors and in accordance with all applicable ethical and legal rules concerning psychological research in the United States of America.

### Procedure

To begin, the child warmed up with two experimenters (the host, who was either a man or a woman, and the adult informant, who was always a woman) in a greeting room with toys. Once the child seemed comfortable, the two experimenters brought the child and their parent to the tea party room. Here, the host labeled the child an ingroup member (by giving the child a blue sticker, which the host and informants also wore, and saying: “We are Duke!”), invited the child to help set up the tea party for an upcoming guest, and told the child that another tea party was occurring in another room. The host then pointed out a laptop that they could use to talk to people in the other room. Next, the adult informant left the room to presumably go to the other room, and the host then pretended to video chat with the adult informant on the laptop. This initial phase, which was meant to convince the child that the video chat was live and real, showed the adult informant with a 6-year-old girl (the child informant) in a similar playroom. In reality, all the footage shown on the laptop was prerecorded. The host then briefly played with a ball with the child before proceeding to the conformity trials.

#### Conformity trials

The tea party room had 4 low shelves, each containing 4 options and 4 instances of each option for one type of tea party item (e.g., the “snack” shelf held 4 donuts, 4 cookies, 4 eggs, and 4 asparagus “veggies”). Tablecloths covered the shelves to prevent the children from handling the items prematurely. Taped on the wall above each shelf were pictures of the 4 options on the shelf.

Each child received 4 conformity trials (1 trial each for the plate, cup, tea, and snack items). On each trial, the host first asked the child which option they felt like using, which children typically indicated by pointing to a picture on the wall. Children’s indications seemed to reflect their actual preferences. When children did not conform, they tended to pick the item they had initially indicated, as described in S1 Text. Next, the host initiated a video chat to check on what was happening in the other room. In a between-subjects design, half of the sample (*n* = 52) video chatted with the adult informant on all conformity trials, whereas the other half (*n* = 52) video chatted with the child informant on all conformity trials. However, both informants followed the same script.

During the video chat, the informant declared that they were looking for an item to use, rejected 3 options for that item, and then endorsed one of the options. To reduce the likelihood that the informant-endorsed option would be one that the children would have liked to use independent of the endorsement, the informant-endorsed option was always one of the less appealing options (e.g., the veggie snack). S1 Text includes more details about the options that were available and which ones were endorsed. In a within-subjects design, the informant gave 2 endorsements framed as norms (“For tea parties at Duke, we always use this kind of snack”) and 2 endorsements framed as preferences (“For my tea party today, I feel like using this snack”). Thus, the norms and preferences differed on several dimensions of conventionality, including reference to the ingroup (“tea parties at Duke” versus “my tea party”), subject (“we” versus “I”), temporal generality (“always” versus “today”), and generic language (“this kind of snack” versus “this snack”).

Next, the host paused the video on a blank frame, emphasized the informant’s endorsement, asked the child to get an item, and removed the tablecloth from the appropriate shelf. The dependent measure was which option the child selected. Children scored 0 (non-conformity) on a trial if they chose any of the 3 non-endorsed options (e.g., the donut, cookie, or egg). In some cases, children did not have the opportunity to conform per our definition because they initially indicated a preference for the option that the informant would later endorse (e.g., the veggie). We reasoned that choosing the endorsed option in such cases did not accurately represent conformity, as the children may have been inclined to select their preferred option independent of the informant’s endorsement.

Thus, the 19 children who initially preferred and subsequently chose the informant-endorsed option on at least one trial scored 0 on such trials. It was advantageous to code such individual trials as 0 rather than exclude the data of these 19 children entirely, since the data from these children’s other trials could still contribute to the overall analysis. Altogether, there were 21 such trials, including 13 in the norm condition (in which the child indicated a preference for an option, heard the informant endorse that option with a norm, and then chose that option) and 8 in the preference condition (in which the child indicated a preference for an option, heard the informant endorse that option with a preference, and then chose that option). Children scored 1 (conformity) on a trial only if they chose the endorsed option after initially indicating a preference for one of the other options. In such cases, children were truly conforming, as they were overriding their own preferences to behave like the informant.

In total, children scored from 0 to 2 in conformity to norms and 0 to 2 in conformity to preferences. For counterbalancing, we crossed order of presentation of item types (plates and cups followed by teas and snacks—or teas and snacks followed by plates and cups) by order of presentation of norms versus preferences (2 norms followed by 2 preferences—or 2 preferences followed by 2 norms). To reduce carryover effects, the host said a transitional remark (e.g., “We’re all done setting up the teas and snacks. In a minute, we’ll set up the plates and cups, okay?”) and played with a ball between the first two and final two conformity trials. Additionally, between each conformity trial, the host briefly distracted the child with a ball. Besides conformity, we also examined a second measure at the end of each session: whether children protested against a puppet who deviated from the informant’s endorsements. Due to the very low rates of protest (6% of the time), we moved discussion of this measure to S2 Text.

## Results

The raw data are available in S1 Table. For interrater reliability, a second coder viewed 23% of the sessions (*n* = 24) and coded which items the child initially preferred and subsequently chose. Agreement was perfect in both respects aside from one ambiguous case in which the child indicated two initial preferences. Using the lme4 package in R version 4.0.0, children’s conformity was analyzed with linear mixed effects models [18], which included random intercepts for participants to account for individual variability rather than treating such variability as error, as in typical regression models, thereby enabling a more powerful analysis. A series of models was created. Model 1 was a null model containing only the random intercept of Participant. Model 2 added the fixed effects of Informant (Child, Adult) and Endorsement (Preference, Norm). Model 3 added the interaction of Informant by Endorsement. Model comparisons using likelihood ratio tests assessed whether each model’s inclusion of additional terms significantly improved the fit to the data. An alpha level of *p* < 0.05 was selected.

The inclusion of the main effects in Model 2 led to a significant improvement in fit compared to Model 1, χ^2^ (2) = 8.59, *p* = 0.01. However, the inclusion of the interaction in Model 3 did not lead to an improvement in fit compared to Model 2, χ^2^ (1) = 0.51, *p* = 0.47, and the interaction was not significant in Model 3 anyways (*b* = -0.12, *SE* = 0.16, *t* = -0.72, *p* = 0.47). Thus, the most parsimonious explanation of the data was Model 2, as shown in Table 1.

**Table 1:** Summary of the linear mixed effects model of conformity as predicted by informant (child, adult) and endorsement (preference, norm).

Formula: Conformity ~ Informant + Endorsement + (1|Participant)					
*Model fit*:	AIC	BIC	logLik	deviance	df.resid
	457.8	474.5	-223.9	447.8	203
*Random effects*:	Variance	Std. Dev.			
Participant	0.2081	0.4562			
Residual	0.3373	0.5808			
*Fixed effects*:	Estimate	Std. Error	df	t value	Pr(>|t|)
(Intercept)	0.2596	0.0942	148.3307	2.757	0.0066**
Informant [Adult]	0.2115	0.1204	104.0000	1.757	0.0818
Endorsement [Norm]	0.1923	0.0805	104.0000	2.388	0.0188*

The Child and Preference conditions were the reference levels.

*p < 0.05

**p < 0.01.

**Fig 1:**
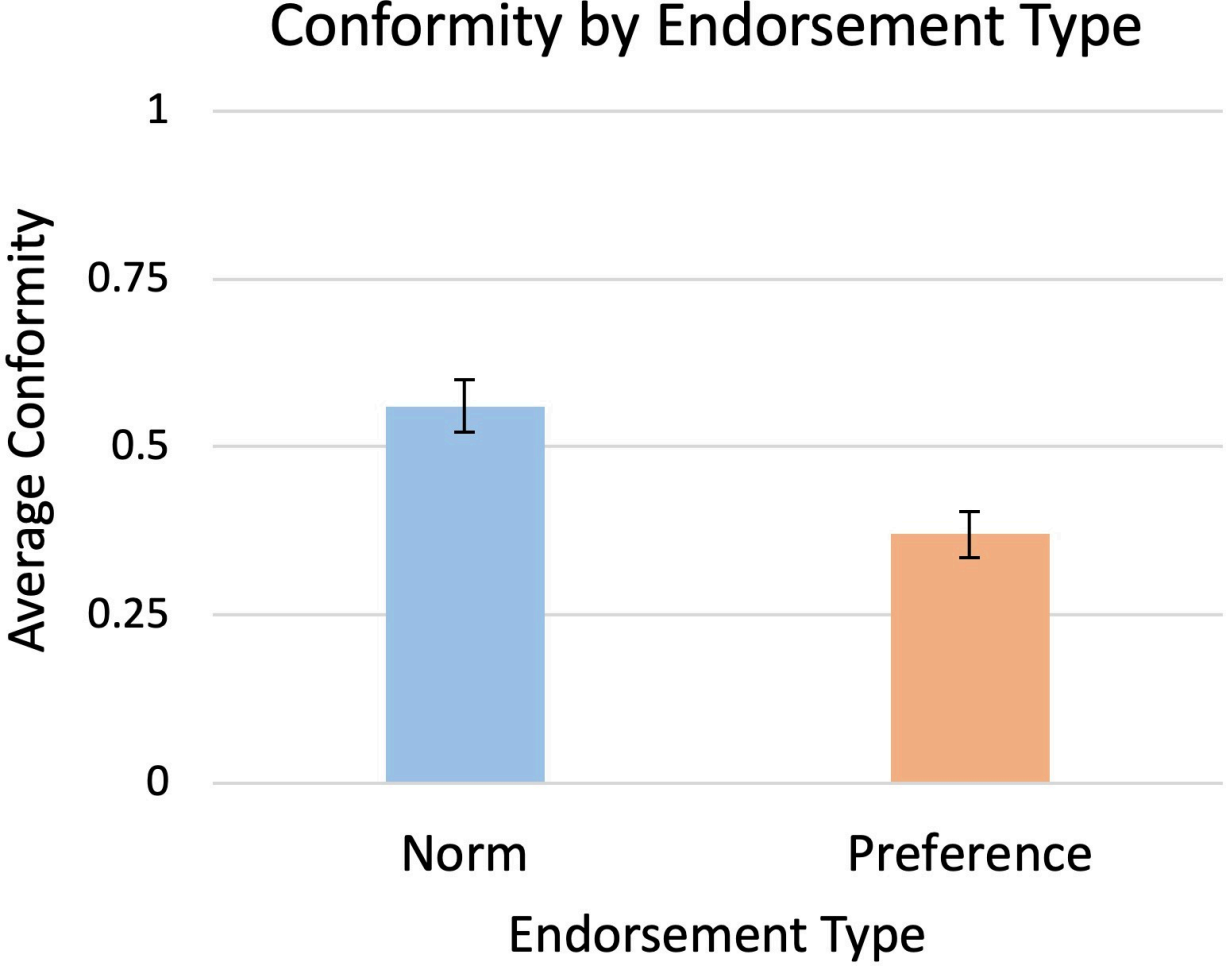
Children conformed more to norms than to preferences. Error bars represent standard errors. Note that the full range of conformity scores (y-axis) is from 0 to 2, although only the range from 0 to 1 is depicted here.

Supporting our hypotheses, Model 2 included a significant main effect of Endorsement (*b* = 0.19, *SE* = 0.08, *t* = 2.39, *p* = 0.02), such that children conformed more to norms (*M* = 0.56, *SD* = 0.80) than to preferences (*M* = 0.37, *SD* = 0.70), as shown in Fig 1. Whereas children’s rate of conformity to norms was higher than expected by chance, χ^2^ (1) = 11.39, *p* = 0.0007, children’s rate of conformity to preferences did not differ from chance, χ^2^ (1) = 0.03, *p* = 0.86. The main effect of Informant was not significant (*b* = 0.21, *SE* = 0.12, *t* = 1.76, *p* = 0.08). That is, children’s conformity to the adult informant (*M* = 1.13, *SD* = 1.36) did not differ significantly from their conformity to the child informant (*M* = 0.71, *SD* = 1.11). S3 Text describes the effects of counterbalancing order, which were all consistent with the results of Model 2 reported here, as well as how the main effect of Endorsement held in both the adult informant and the child informant conditions separately.

Given the equivocal *p* value of the main effect of Informant (*p* = 0.08), we conducted further analyses in G*Power [19] to assess how much power our study had to detect (or rule out) a potential effect of Informant. A sensitivity analysis showed that a study with our parameters (alpha = 0.05, sample size = 104, numerator df = 1, number of groups = 4) could have detected an effect size of *f* = 0.28 (slightly larger than a conventionally medium effect size) with a power of 0.8. Moreover, a power analysis showed that a study with our parameters (alpha = 0.05, numerator df = 1, number of groups = 4) would have required a sample size of 128 participants to detect a conventionally medium effect size (*f* = 0.25) with a power of 0.8. For smaller effect sizes, even larger sample sizes would have been required. Thus, our study may have lacked the power to detect (or conclusively rule out) a potential effect of Informant, so our findings should not be taken as strong evidence either for or against a potential effect of Informant. Given the ambiguities about how to interpret *p* values greater than 0.05—and given that a main effect of Informant would be independent of our main hypotheses in any case—we elected to refrain from drawing further conclusions about the potential effect of Informant.

## Discussion

In a pretend tea party, 3.5-year-old children first indicated which items they preferred to use but then heard another person, who was either an adult or another child, give endorsements of other items. Children overrode their own preferences and conformed to the other person more when the endorsements were framed as conventional norms than when they were framed as personal preferences. Moreover, children conformed more to norms than to preferences whether the informant was an adult or a child, suggesting that it is conventionality, not just adult authority, that matters. Although the children in our norm condition conformed relatively infrequently (28%), this conformity rate was consistent with the conformity rate (also 28%) in a previous study of children’s conformity [11]. The relatively low conformity rate was unsurprising, given that choosing to conform meant going against one’s own preferences. Considering the subtle linguistic framing that we used, which specified norms in terms of what group members descriptively “always” do instead of what they prescriptively “should” do, it is noteworthy that children still overrode their own preferences to conform.

Our findings complement previous evidence and arguments suggesting that children are motivated to act conventionally [4, 6–8, 10, 11, 20]. Such a motivation to act conventionally would have been adaptive throughout human evolution. It would have helped children navigate important social challenges, such as affiliating with one’s cultural group, learning causally opaque but meaningful social practices, and selectively learning which actions performed by others are necessary to adopt (e.g., social norms) and which are not (e.g., others’ preferences). Given its many functions, such a motivation to act conventionally would have contributed substantially to the development of human culture and human uniqueness.

Humans inherit from their forebears not only their genes but also their cultural practices. For this process of cultural inheritance to work, younger generations must be motivated to acquire culture from older generations. Previous studies have suggested that young children are motivated to act conventionally, but these studies were limited in that they did not rule out plausible alternative explanations for why children may seek to act in conventional ways (e.g., perhaps children only perform conventional actions because they seek to affiliate with others or achieve instrumental goals). By using a task setting that accounted for these alternative explanations, we provided further evidence that young children do have a specific motivation to act conventionally.

In our study, conformity was the dependent measure of interest, but researchers have also used other kinds of dependent measures to probe children’s developing understanding of norms. For instance, a large body of research has examined how children react when others violate norms. This line of research has revealed that children protest against transgressors [21] and tattle on transgressors to observers [22]. Such acts of third-party norm enforcement, in which children intervene against transgressions that do not personally harm them, indicate that children are committed to upholding norms above and beyond simply protecting their own self-interest.

Other research has examined whether children can create new norms during peer interactions. An ability to create new norms with peers, not just follow existing norms handed down by adults, is significant because it speaks to an understanding that norms are essentially social agreements. This line of research has shown that 5-year-olds can create novel norms with peers [23, 24]. Moreover, 5-year-olds also teach their self-created norms to novices [23, 24]. In future research, it may be worthwhile to examine whether a priority of norms over preferences would manifest not only in the dependent measure of conformity but also in other relevant dependent measures, such as protesting, tattling, norm creation, and norm teaching. Plausibly, children may protest and tattle more against deviations from norms than against deviations from preferences (although, as we note in S2 Text, we observed little protest in our study). It would also be interesting to analyze children’s discourses during norm creation and transmission to see how children themselves linguistically cue conventionality.

There are some limitations of our study to consider. One limitation of our study was that the endorsements of both the adult informant and the child informant were emphasized by the adult host. Thus, in both cases, participants may have felt that the informant’s endorsements were bolstered by adult authority. This methodological concern may be tempered by the fact that the host always asked the adult/child informant what they were up to and followed the informant’s lead by emphasizing what the informant said, so the source of the endorsement was known to be the informant, not the host. Nonetheless, this may merit future research with a more controlled design.

A second methodological limitation was that we did not assess the strength of the children’s own preferences beyond simply asking children to indicate which option they felt like using at the beginning of each trial. Potentially, children’s willingness to conform (i.e., their willingness to override their own preference and adopt someone else’s choice) may have varied across trials based on how committed the children were to their own preferences on particular trials. In future research, it may be interesting to examine whether the influence of linguistic cues on children’s behaviors would vary depending on the strength of the children’s own preferences. A third limitation was that the child informant, a 6-year-old girl, was older than our 3.5-year-old participants. To our participants, the child informant may have actually exuded authority, so our conclusion that children prioritized norms out of respect for conventionality, not just respect for authority, invites further investigation. Future research could also examine whether children consider same-age or even younger peers to be valid messengers of norms.

A fourth limitation was that the expressions of norms and preferences differed on several dimensions, not just one. As this was the first study (to our knowledge) to compare the relative effects of norms and preferences on children’s conformity, we elected to present norms and preferences as they occur naturalistically—with multiple features differing between them—to first establish whether they differed from each other as a whole. To repeat, the norms were expressed by saying: “For tea parties at Duke, we always use this kind of (item).” The preferences were expressed by saying: “For my tea party today, I feel like using this (item).” As such, the norms and preferences differed by several features, including reference to the ingroup, subject, temporal generality, and use of generic language.

Thus, although we found a main effect of endorsement type, such that children conformed more to norms than to preferences, it remains unclear which particular features of norms were the ones responsible for promoting conformity. It is likely that multiple features, not just one, had an influence on children’s behavior, but additional research will be needed to disentangle their effects. Particularly, more research should examine linguistic cues of group membership and whether such cues are effective at influencing children’s behaviors. One relevant study found that children interpret descriptive cues about how members of a group regularly behave to be, indeed, prescriptive cues for how members of that group should behave [25]. In other words, children make an inferential leap from descriptive information (how group members regularly act) to prescriptive expectations (how group members should act) [25]. Further research into how children interpret and respond to cues of group membership could help test the influential hypothesis [4, 8, 15, 20] that respect for the group is a powerful source of normativity for young children.

As for our study, our results were certainly consistent with the hypothesis that respect for the group is a source of normativity for young children. However, we acknowledge that we cannot conclusively assert that it was respect for the group and not some other possible feature (e.g., the temporal generality or the use of generic language in the phrasing) that led children to favor norms over preferences in our study, so further research is warranted. In future studies focusing on whether groups are perceived as sources of normativity by children, it may be advisable for researchers to assess children’s reactions to being labeled as a new member of the group, such as by asking children about their attitudes towards the group.

Finally, a fifth limitation was that our sample consisted of relatively affluent children in a Western context, so our conclusions warrant further study from broader cultural contexts. Notably, previous research found that framing a necklace making activity as conventional rather than instrumental increased imitative fidelity not only for Western children from the United States but also for non-Western children from Melanesia, suggesting universal processes [1]. In addition to assessing how non-Western children interpret and respond to linguistic cues of conventionality, future research may also address more targeted questions regarding, for instance, the magnitudes of such effects in different cultures or the age at which children from different cultures begin to prioritize conventionality. Given that human uniqueness is based in large part on the human capacity for culture, further research on how young children from different cultures acquire their various cultural competencies will go a long way towards answering the timeless question of how human psychology became unique.

## Supporting information

S1 Text Children’s initial preferences and subsequent choices. (DOCX)

S2 Text Protest measure. (DOCX)

S3 Text Additional analyses. (DOCX)

S1 Table Raw data. (CSV)
