# Supplementary material for: Young children conform more to norms than to preferences
Source: PLoS One. 2021 May 26;16(5):e0251228. doi: 10.1371/journal.pone.0251228 (PMC8153413; doi:10.1371/journal.pone.0251228)
Supplement: S1 Text — (DOCX) [file pone.0251228.s001.docx]

**Children’s Initial Preferences and Subsequent Choices**

According to the data, children’s indications of which items they felt like using seemed to correspond to their actual preferences. For each item option, we report the number of children who initially indicated that they felt like using that option as well as the number and percentage of those children who actually chose that option, consistent with their indication. For instance, 8 children indicated that they felt like using the zebra plate. Of those 8 children, 5 children (63%) actually chose the zebra plate. Children chose what they indicated 62% of the time, which was a majority of the time and higher than expected at a chance level of 25%, χ^2^ (1) = 304.05, *p* < 0.01. Item options are listed in the order in which they were presented by the informant.

| Option (* indicates option endorsed by informant) | # of children who indicated a preference for the option | # of children who chose what they indicated (per option) | % of children who chose what they indicated (per option) |
| --- | --- | --- | --- |
| **Plates** | | | |
| zebra | 8 | 5 | 63% |
| rainbow | 83 | 62 | 75% |
| round white | 10 | 6 | 60% |
| square white* | 2 | 2 | 100% |
| forgot to ask | 1 | N/A | N/A |
| **Cups** | | | |
| smiley face | 26 | 10 | 38% |
| Frozen | 52 | 36 | 69% |
| red | 12 | 4 | 33% |
| blue* | 14 | 11 | 79% |
| **Teas** | | | |
| apple | 56 | 37 | 66% |
| orange | 31 | 18 | 58% |
| celery | 8 | 2 | 25% |
| potato* | 9 | 6 | 67% |
| **Snacks** | | | |
| donut | 48 | 35 | 73% |
| cookie | 41 | 18 | 44% |
| egg | 11 | 4 | 36% |
| veggie* | 4 | 2 | 50% |
