# Supplementary material for: Young children conform more to norms than to preferences
Source: PLoS One. 2021 May 26;16(5):e0251228. doi: 10.1371/journal.pone.0251228 (PMC8153413; doi:10.1371/journal.pone.0251228)
Supplement: S2 Text — (DOCX) [file pone.0251228.s002.docx]

**Protest Measure**

**Method**

In addition to the conformity measure, our study also included a second measure assessing whether children would protest when a squirrel puppet deviated from the informant’s endorsements. The host first introduced the child to the squirrel puppet during their warm-up in the greeting room prior to going to the tea party room together. Later, the assessment of protest occurred after all the conformity trials were completed. During this protest phase, the host informed the child that a squirrel puppet (operated by the host) would also set up for the tea party. The puppet then chose items that differed from the informant’s prior endorsements (as well as from the child’s choices in cases in which the child had deviated from the informant’s endorsement). The puppet gave the child two opportunities to object—first by saying, “Maybe I’ll use this one. . .” and then, if the child had not objected, “Should I use this one?”

A score of 0 (representing no protest) was given if the child did not protest. Children also scored 0 if they protested but endorsed an item other than what the informant had endorsed. We reasoned that such cases may not accurately represent protest in service of upholding a norm, since the child could have simply wanted the puppet to fulfill their own preferences. A score of 1 (representing protest) was given only if the child advised the squirrel puppet to choose the item that the informant had endorsed.

## **Results**

For inter-rater reliability, a second coder viewed 25% of the sessions (*n* = 26) and coded whether the child protested or not. This second coder had 100% agreement with the previously coded data. Rates of protest were very low. In only 23 instances (6% of 416 possible opportunities to protest) did children advise the puppet to choose the item that the informant had endorsed. These 23 cases included 16 protests against deviations from norms and 7 protests against deviations from preferences. The difference between the number of protests against deviations from norms and the number of protests against deviations from preferences was not significant, as indicated by a two-tailed binomial test, *p* = 0.09.

**Discussion**

Overall, our protest measure appeared to have not worked, given that children rarely protested. This finding was not too unexpected, given that our task manipulation was a subtle linguistic framing. It was interesting, then, that the same linguistic cues that were not strong enough to lead children to correct how others behave were nonetheless strong enough to sway children’s own behavior. Here, we discuss three plausible interpretations for why protest behaviors were low compared to in previous studies. One interpretation of this finding is that the perceived force of a norm may reside on a gradient, such that some norms exert enough pressure that one feels compelled to follow them but not enough pressure that one feels compelled to enforce them on others.

A second interpretation pertains to children’s memory capacities. Children encountered the protest phase after having experienced four conformity trials in which the informant endorsed one of four options for a tea party item. That is, children observed the informant endorse four of sixteen different options for the tea party over four different trials. It is possible that by the time children encountered the protest phase, they did not clearly recall which items the informant had endorsed, let alone which items the informant had endorsed with norms and which with preferences. Future research could potentially reduce memory demands by presenting individual protest trials after individual conformity trials rather than together in a block at the end of the study.

A third interpretation pertains to a more practical—but, in our view, not insignificant—aspect of our study design. In most studies that include protest against a puppet as a dependent measure, there is a warm-up phase in which a puppet acts in ways that invite intervention (e.g., in a clumsy or clearly erroneous manner). In this warm-up phase, children have the opportunity to practice correcting the puppet. Accordingly, by the time children encounter the test phase, they are primed to think of the puppet as someone they can object to. However, our warm-up phase did not involve the puppet acting in ways that invited intervention. In our warm-up phase, the puppet simply introduced themselves to the child. As a result, the children in our study may have been less prepared—compared to children in other studies—to object to the puppet when it acted in ways that deviated from the endorsements of the informant. Other researchers may learn from our experience and be sure to include a warm-up phase to encourage higher rates of protesting.
