# Supplementary material for: Young children conform more to norms than to preferences
Source: PLoS One. 2021 May 26;16(5):e0251228. doi: 10.1371/journal.pone.0251228 (PMC8153413; doi:10.1371/journal.pone.0251228)
Supplement: S3 Text — (DOCX) [file pone.0251228.s003.docx]

**Additional Analyses**

We conducted additional exploratory analyses to examine (i) the possible effects of counterbalancing Order (Preference First, Norm First) and (ii) whether the main effect of Endorsement held in both the adult informant and the child informant conditions separately. Overall, all analyses were consistent with the results that we reported in our main text. Order did have a significant interaction with Endorsement, but it was in a way that was still consistent with our interpretation of the main effect of Endorsement. Most importantly, Order did not interact with Informant and did not alter the (lack of an) interaction between Informant and Endorsement. In other words, our main conclusion (that children conformed more to norms than to preferences whether the informant was an adult or a child) remained intact even after accounting for the effects of Order. Moreover, the main effect of Endorsement was significant in both the adult informant and the child informant conditions separately.

**Main Effect of Order**

To assess the effects of Order, we ran and compared a series of models. Our baseline model that served as a basis for model comparison was a null model that only included the random intercept of Participant. The simplest model of interest added the main effect of Order to the null model. The condition “Preference First” was chosen as the reference level for Order, as we expected a priori that children would conform less in this case. However, this model containing the main effect of Order did not lead to a significant improvement in fit compared to the null model, χ^2^ (1) = 2.59, *p* = 0.11, and also did not find a significant main effect of Order (*b* = 0.20, *SE* = 0.12, *t* = 1.62, *p* = 0.11).

**Crossing Order and Informant**

In a second model, we examined whether Order interacted with Informant. In this model, we added the main effects of Order and Informant as well as their interaction to the null model. However, this model also did not lead to a significant improvement in fit compared to the null model, χ^2^ (3) = 6.02, *p* = 0.11. What is more, this model showed no significant main effect of Order (*b* = 0.24, *SE* = 0.17, *t* = 1.40, *p* = 0.17), no significant main effect of Informant (*b* = 0.25, *SE* = 0.17, *t* = 1.47, *p* = 0.14), and no interaction between Order and Informant (*b* = -0.06, *SE* = 0.24, *t* = -0.26, *p* = 0.79).

**Crossing Order and Endorsement**

In a third model, we examined whether Order would interact with Endorsement. Thus, in this model, we added the main effects of Order and Endorsement as well as their interaction to the null model (Table 1a). Unlike the first two models, this model did lead to a significant improvement in fit compared to the null model, χ^2^ (3) = 12.24, *p* = 0.007. There was a significant main effect of Order (*b* = 0.36, *SE* = 0.14, *t* = 2.47, *p* = 0.01): Children conformed more when they heard norms first than when they heard preferences first. There was also a significant main effect of Endorsement (*b* = 0.36, *SE* = 0.11, *t* = 3.16, *p* = 0.002): Children conformed more to norms than to preferences. These main effects were qualified by a significant interaction between Order and Endorsement (*b* = -0.32, *SE* = 0.16, *t* = -2.04, *p* = 0.04).

The interaction revealed that when children heard norms first, they conformed at similar rates to the norms (*M* = 0.57) as to the subsequent preferences (*M* = 0.54). But when children heard preferences first, they conformed more to norms (*M* = 0.54) than to preferences (*M* = 0.18). To confirm whether the difference between conformity to norms and conformity to preferences was significant within the subset of children who heard preferences first, we ran another model examining only the main effect of Endorsement in the subset of children who had preferences first (Table 1b). This model led to a significant improvement in fit compared to a null model that only included the random intercept of participant (for the subset of children who had preferences first), χ^2^ (1) = 11.31, *p* = 0.0008, and also confirmed that the main effect of Endorsement was significant within this subset (*b* = 0.36, *SE* = 0.10, *t* = 3.56, *p* = 0.0008).

Importantly, the interaction between Order and Endorsement was still consistent with our hypothesis that children are motivated to conform in response to cues of conventionality. That is, hearing norms first may have led children to construe the entire play situation as normative, including the preferences that were stated later. In such cases, when children heard the preferences stated by the informant who had previously established themselves as a cultural representative, they may have interpreted the informant’s preferences to also be expressions of norms. But when children heard preferences first and norms only later, they may not have begun to construe the situation as normative until after they had freely acted on their own preferences. Hence, conformity to norms was greater than conformity to preferences within this latter subset.

|  |  |  |  |  |  |  |  |
| --- | --- | --- | --- | --- | --- | --- | --- |
|  | A. Formula: Conformity ~ Order * Endorsement + (1\|Participant) | | | | | |  |
|  |  |  |  |  |  |  |  |
|  | *Model fit:* | AIC | BIC | logLik | deviance | df.resid |  |
|  |  | 456.2 | 476.2 | -222.1 | 444.2 | 202 |  |
|  |  |  |  |  |  |  |  |
|  | *Random effects:* | Variance | Std. Dev. |  |  |  |  |
|  | Participant | 0.2163 | 0.4650 |  |  |  |  |
|  | Residual | 0.3243 | 0.5694 |  |  |  |  |
|  |  |  |  |  |  |  |  |
|  | *Fixed effects:* | Estimate | Std. Error | df | t value | Pr(>\|t\|) |  |
|  | (Intercept) | 0.1800 | 0.1040 | 179.2973 | 1.731 | 0.0851 |  |
|  | Order [Norm] | 0.3570 | 0.1443 | 179.2973 | 2.474 | 0.0143* |  |
|  | Endorsement [Norm] | 0.3600 | 0.1139 | 104.0000 | 3.161 | 0.0021** |  |
|  | Order [Norm] x | -0.3230 | 0.1581 | 104.0000 | -2.043 | 0.0435* |  |
|  | Endorsement [Norm] |  |  |  |  |  |  |
|  |  |  |  |  |  |  |  |
|  |  |  |  |  |  |  |  |
|  | B. Formula: Conformity ~ Endorsement + (1\|Participant) | | | | | |  |
|  |  |  |  |  |  |  |  |
|  | *Model fit:* | AIC | BIC | logLik | deviance | df.resid |  |
|  |  | 202.8 | 213.2 | -97.4 | 194.8 | 96 |  |
|  |  |  |  |  |  |  |  |
|  | *Random effects:* | Variance | Std. Dev. |  |  |  |  |
|  | Participant | 0.2028 | 0.4503 |  |  |  |  |
|  | Residual | 0.2552 | 0.5052 |  |  |  |  |
|  |  |  |  |  |  |  |  |
|  | *Fixed effects:* | Estimate | Std. Error | df | t value | Pr(>\|t\|) |  |
|  | (Intercept) | 0.1800 | 0.0957 | 83.6073 | 1.881 | 0.0635 |  |
|  | Endorsement [Norm] | 0.3600 | 0.1010 | 50.0000 | 3.563 | 0.0008** |  |
|  |  |  |  |  |  |  |  |

Table 1. Panel A: Summary of the linear mixed effects model of conformity as predicted by Order (Preference First, Norm First) crossed with Endorsement (Preference, Norm). Panel B: Summary of the linear mixed effects model of conformity as predicted by Endorsement (Preference, Norm) within the subset of children who heard preferences first. **p* < 0.05; ***p* < 0.01.

**Crossing Order, Informant, and Endorsement**

In a fourth model, we examined whether the (lack of an) interaction between Informant and Endorsement would still hold after accounting for Order. This was important to verify, as one of our key conclusions was that children conformed more to norms than to preferences whether the informant was an adult or a child. Thus, we included the main effects and interactions of all three variables: Order, Informant, and Endorsement. However, this fourth model did not lead to a significant improvement in fit compared to our third model (i.e., the model crossing Order and Endorsement), χ^2^ (4) = 4.22, *p* = 0.38, so our third model remained the most parsimonious explanation of the data.

Nonetheless, the results of this fourth model were still consistent with our conclusions. The only significant effects in this model were a main effect of Order (*b* = 0.43, *SE* = 0.20, *t* = 2.12, *p* = 0.04) and a main effect of Endorsement (*b* = 0.46, *SE* = 0.16, *t* = 2.80, *p* = 0.006). Importantly, the interaction between Informant and Endorsement was not significant (*b* = -0.19, *SE* = 0.23, *t* = -0.83, *p* = 0.41), and the interaction between Order, Informant, and Endorsement was also not significant (*b* = 0.12, *SE* = 0.32, *t* = 0.37, *p* = 0.71). Thus, even when accounting for Order and its interactions with the other variables, our main conclusion remained intact: Children conformed more to norms than to preferences whether the informant was an adult or a child.

**The Adult and Child Informant Conditions**

A final question of interest was whether the main effect of Endorsement would hold in the separate subsets of children who heard from the adult and child informants. Given that the main effect of Endorsement was found to be qualified by an interaction between Order and Endorsement in our third model, we limited our analyses to the subset of children who heard preferences first, since the subset of children who heard norms first had restricted variability in the differences between their rates of conformity to the norms and the preferences. Focusing on the subset of children who had the adult informant and preferences first, we ran a model that only included the main effect of Endorsement (Table 2). This model led to a significant improvement in fit compared to a null model that only included the random intercept of participant (for this subset of children), χ^2^ (1) = 4.07, *p* = 0.04, and also confirmed that the main effect of Endorsement was significant within this subset (*b* = 0.27, *SE* = 0.13, *t* = 2.10, *p* = 0.046). Namely, these children conformed more to norms (*M* = 0.62) than to preferences (*M* = 0.35).

|  |  |  |  |  |  |  |  |
| --- | --- | --- | --- | --- | --- | --- | --- |
|  | Formula: Conformity ~ Endorsement + (1\|Participant) | | | | | |  |
|  |  |  |  |  |  |  |  |
|  | *Model fit:* | AIC | BIC | logLik | deviance | df.resid |  |
|  |  | 113.9 | 121.7 | -52.9 | 105.9 | 48 |  |
|  |  |  |  |  |  |  |  |
|  | *Random effects:* | Variance | Std. Dev. |  |  |  |  |
|  | Participant | 0.3639 | 0.6032 |  |  |  |  |
|  | Residual | 0.2138 | 0.4623 |  |  |  |  |
|  |  |  |  |  |  |  |  |
|  | *Fixed effects:* | Estimate | Std. Error | df | t value | Pr(>\|t\|) |  |
|  | (Intercept) | 0.3462 | 0.1491 | 37.2266 | 2.322 | 0.0258* |  |
|  | Endorsement [Norm] | 0.2692 | 0.1282 | 26.0000 | 2.100 | 0.0456* |  |
|  |  |  |  |  |  |  |  |

Table 2. Summary of the linear mixed effects model of conformity as predicted by Endorsement (Preference, Norm) within the subset of children who heard preferences first from the adult informant. **p* < 0.05; ***p* < 0.01.

Next, focusing on the subset of children who had the child informant and preferences first, we ran a model that only included the main effect of Endorsement. However, the model failed to converge due to having an insufficient number of observations. Whereas in the adult informant condition, our sample included a perfect balance of children who heard preferences first (*n* = 26) and children who heard norms first (*n* = 26), in the child informant condition, we had slightly fewer children who heard preferences first (*n* = 24) than children who heard norms first (*n* = 28) due to imperfect counterbalancing during recruitment. Thus, in lieu of running a model, we conducted a paired-samples Wilcoxon signed-rank test instead. This Wilcoxon signed-rank test nonetheless confirmed that children in this subset conformed significantly more to norms (*M* = 0.46) than to preferences (*M* = 0.00), *Z* = -2.34, *p* = 0.02, *r* = 0.34. Indeed, the children in this subset actually never conformed to the preferences at all.
